# Regeneration of the mammalian brain: a relic of evolution?

**DOI:** 10.1093/stmcls/sxag029

**Published:** 2026-05-12

**Authors:** Olena Zhulyn, Vanessa Donega

**Affiliations:** Developmental, Stem Cell & Cancer Biology Program, SickKids Research Institute, Toronto, M5G 0A4, Canada; Department of Molecular Genetics, University of Toronto, Toronto, M5G 0A4, Canada; Department of Anatomy and Neurosciences, Amsterdam UMC location Vrije Universiteit Amsterdam, Amsterdam, 1081 HZ, The Netherlands; Amsterdam Neuroscience, Cellular and Molecular Mechanisms, Amsterdam, The Netherlands

**Keywords:** mammalian regeneration, adult neurogenesis, stem cells, brain damage

## Abstract

Compared to amphibians, fish, and reptiles, mammals have an impaired capacity to regenerate the brain, despite the presence of a neural stem cell pool. Here, we consider the biological significance of a stem cell source in the human brain by exploring evolutionary trade-offs that may guide retention of regenerative capacity in mammals and the risks and benefits of active neurogenesis in adults. We discuss whether reduced regenerative capacity in humans is adaptive or stochastic and examine the role of the injured and diseased brain environment in preventing efficient brain repair. We discuss the therapeutic potential of activating the latent regenerative potential of the human brain to reverse tissue damage or degeneration and whether this approach carries with it an unanticipated risk of cancer.

## Graphical abstract

**Graphical Abstract:**
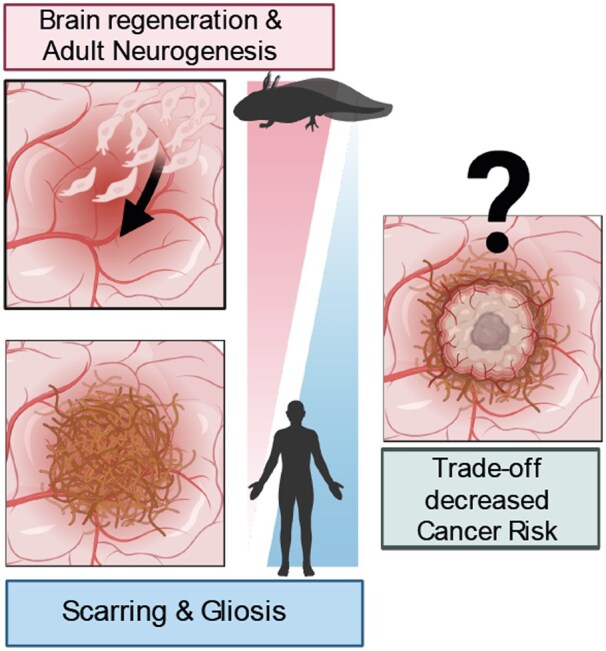

Significance statement Mammalian species have an impaired capacity to regenerate the brain, despite the presence of a neural stem cell pool. In this review, we consider the biological significance of a stem cell source in the mammalian brain. We place the capacity of the human brain to regenerate within an evolutionary context and discuss the significance of regeneration in tumor formation, and its implications for the capacity of the human brain to regenerate. Determining whether regeneration of the mammalian brain is an evolutionary relic or a remnant of evolution that can be triggered if properly stimulated is a crucial step in the development of novel therapeutic strategies.

## Introduction

**Figure 1.**
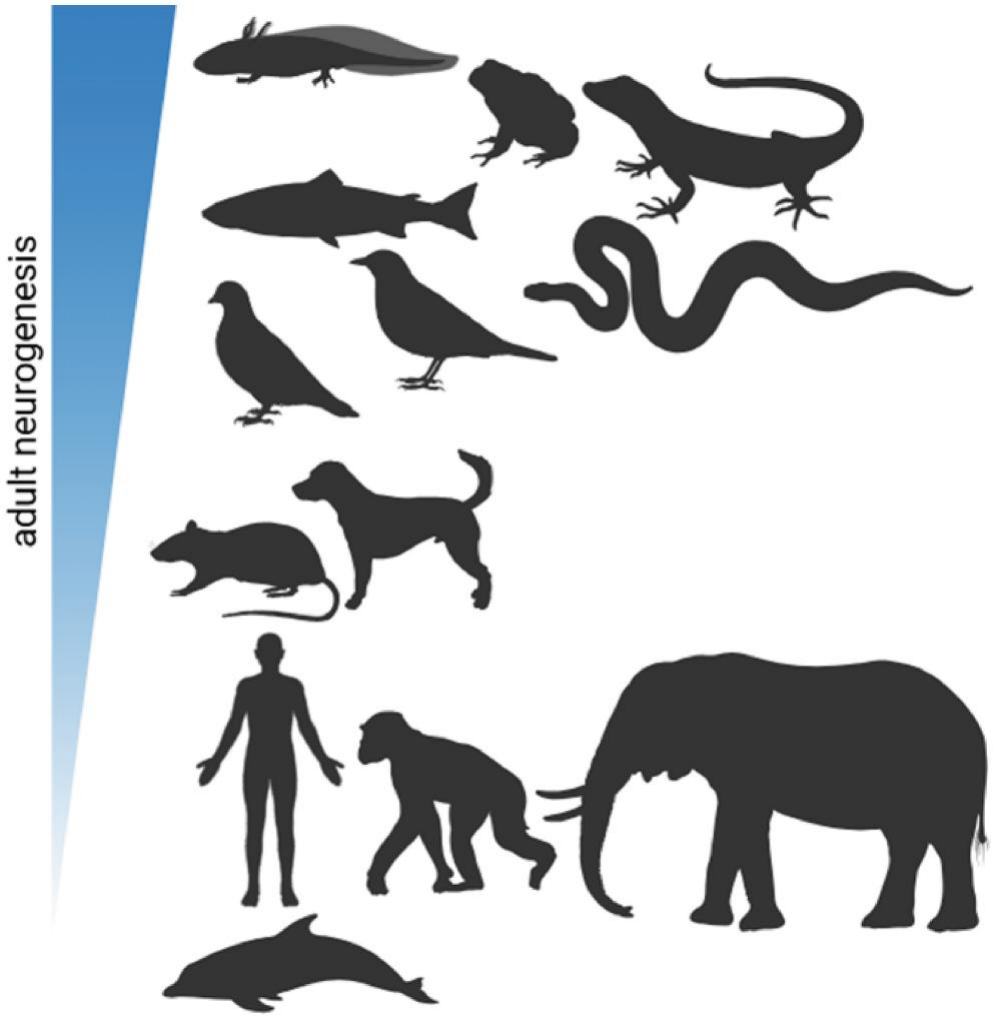
Adult neurogenesis across vertebrates. Although species-specific data are limited, analysis of the literature suggests that robust adult neurogenesis persists across amphibians, reptiles, fish, and birds with mammals generally exhibiting a steep decline in this process, particularly with aging. Among mammals, adult neurogenesis remains best studied and appears to be most pronounced in smaller species including mice, rats, and other rodents. Conversely, “ large-brained” mammals including elephants, humans, and other primates exhibit impaired adult neurogenesis. Studies suggest that this process is further reduced among aquatic mammals such as dolphins and whales.^1–4^ Figure made in BioRender.

Damage to the adult brain, due to injury or neurodegeneration, is a source of significant mortality and morbidity worldwide. While the human brain has impaired regenerative potential, other vertebrates retain the ability to regenerate the brain (Figure 1).^1–4^ To achieve this, animals rely on *neurogenesis*, that is the production of new neurons from neural stem cells (NSCs), which differentiate, migrate, and integrate into the correct brain region.

**Figure 2.**
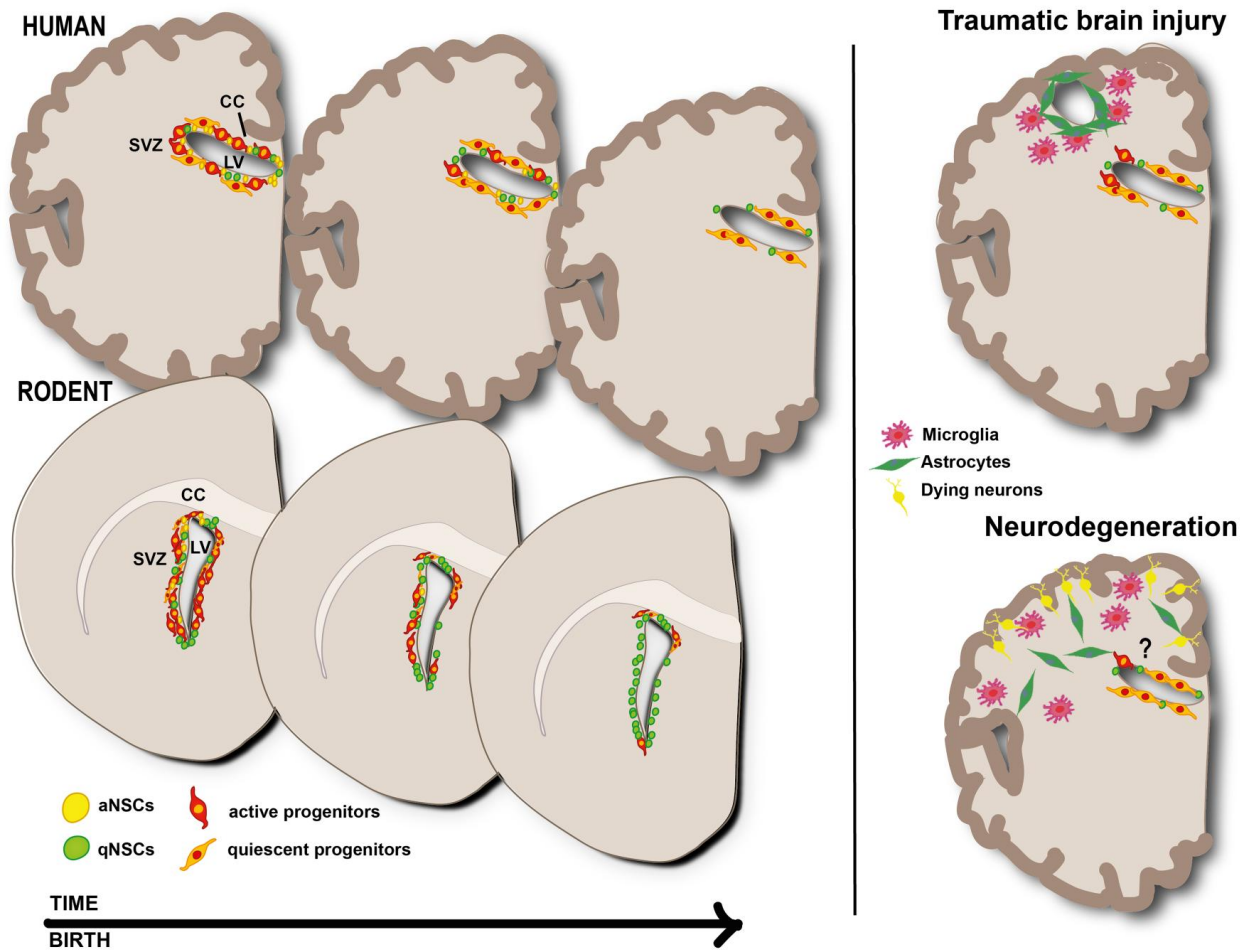
Adult neurogenesis in mammals. Schematic overview of a coronal section of a mouse and human brain showing the SVZ aligning the lateral ventricle. During aging, the number of NSCs and progenitors decreases, as does the number of active proliferating NSCs. In contrast, the number of quiescent NSCs increases with aging. Despite aging or disease, a number of studies in humans have shown that NSCs are not depleted from the SVZ. SVZ = subventricular zone; LV = lateral ventricle; CC = corpus callosum.

Adult neurogenesis in the mammalian brain was first described in the 60s by groundbreaking work from Altman and Das that found evidence for proliferating stem cells in the brain of adult rats and guinea pigs.^5–7^ Now several decades later, two stem cell pools, the so called neurogenic regions, have been identified in the mammalian brain, the subgranular zone (SGZ) in the dentate gyrus of the hippocampus and the subventricular zone (SVZ) aligning the lateral ventricles in the brain.^5^^,^^8–10^ In rodents, under homeostatic conditions, new neurons generated in the SVZ migrate to the olfactory bulb, which is critical for olfactory learning and discrimination.^11^ The SGZ produces new functional neurons that integrate into the hippocampus, a process essential for learning and memory.^12^ In humans, on the other hand, while neurogenesis in the SGZ continues throughout adulthood, there is a steep decline with aging starting from early childhood.^1^^,^^13–16^ The addition of new neurons to the olfactory bulb from the SVZ is likely negligible,^14^^,^^17^^,^^18^ and the few new neurons produced in the SVZ migrate to the neighboring striatum^14^. In the mammalian brain, NSCs become increasingly quiescent with aging.^19^^,^^20^ In mice, this was shown to correlate with an increase in inflammatory factors and a decrease in the activation of the Wnt signaling pathway, which is essential for neurogenesis (Figure 2).^19^ For recent reviews discussing adult neurogenesis in the mammalian brain and the effect of aging on stem cell biology, please see Chaker et al.^12,21^ and Rando et al.^22^

In this perspective, we will discuss the biological significance of a stem cell source in the mammalian brain. We place the impaired capacity of the human brain to regenerate within an evolutionary context and discuss whether this impaired regenerative capacity reflects a safeguard to prevent tumor formation. Finally, we also explore the role of the injured and diseased brain environment in regulating the potential of the mammalian brain to regenerate.

## Regeneration: rising from the ashes

Following tissue development, some tissue types require continuous regulation of their size and cell renewal to ensure normal function and adaptation to external factors. This process of tissue homeostasis occurs throughout life and is dependent on tissue resident stem cell pools to renew and replace potentially damaged cells. The skin,^23^ the intestinal epithelium,^24^ and liver^25^ undergo continuous renewal of cells for tissue maintenance and are among the most regenerative tissues in humans. In contrast, the human brain is among the least regenerative tissue and contains a quiescent population of NSCs which in other species are mobilized for regeneration.

Regeneration, that is, the ability to restore a functional body part, has fascinated scientists since the 1700s. Since then, it has been shown that animal species differ dramatically in their ability to regenerate. Indeed, regeneration can range in scope from repair of cellular extensions (e.g. muscle fibers or neuronal fibers called axons) to restoration of full organs (e.g. limb or spinal cord regeneration in salamanders).^26^ In the most extreme cases, species like planarians (flatworms of the class Turbellaria) can restore their entire body from a small fragment.^27^

Regeneration can be classified into two types: epimorphic regeneration in which tissues are replaced through blastema-mediated growth (i.e. as seen in salamander limbs or lizard tails) and morphallaxis whereby existing tissue is reorganized and repatterned.^26^ Compensatory regeneration can occur through proliferation of existing stem cells, for example, as observed in hepatocytes which proliferate in liver regeneration or stem cell–mediated regeneration in tissues like the skin.^23^^,^^25^ While these processes often occur in response to injury, “physiological” regeneration whereby tissues undergo constant replenishment, often facilitated by specialized stem cell niches, is observed in tissues with high or regular turnover including skin or intestinal epithelium.^23^^,^^24^

Although the prevalence of tissue specific regeneration is highly correlated with overall regenerative capacity, different tissues may leverage distinct mechanisms for self-renewal and regeneration even within the same animal. For example, species like the axolotl (an aquatic salamander) and spiny mouse exhibit high regenerative capacity overall and extensive neurogenesis.^27^^,^^28^ In the axolotl regeneration of the brain is driven by ependymoglial cells (EGCs). Upon injury, the EGCs give rise to a diversity of cell types that integrate into the existing brain circuitry to restore function.^29–31^ The spiny mouse (genus: *Acomys*) has the ability to regenerate the skin, ear, and skeletal muscle.^28^ This entails that multiple tissue types have to regenerate, e.g. injury to the ear is fully repaired with the growth of new blood vessels, cartilage, muscle, skin, and nerve fibers. Interestingly, neither the Muridae subfamily *Rattus* nor *Mus Musculus* has this enhanced regenerative capacity.^32^ It has been suggested that the regenerative capacity of spiny mice is an adaptive mechanism that allows this species to shed and heal its skin thereby escaping predators.^33^

It is unclear why the capacity of animals to regenerate differs between phylogeny. The ability to regenerate at least some tissue types is common and is dispersed among different animal groups.^26^ If regeneration was an epiphenomenon, that is of ancestral origin that originated as a byproduct of development, one would expect it to be widespread among different animal groups.^26^ Another theory is that it is an adaptive feature to the environment (e.g. sublethal predation), and that for some animal species, it is part of a survival mechanism, such as in salamanders which can re-grow a new limb or tail following injury or amputation.^26^^,^^34^ It has also been suggested that regeneration may be a *de novo* feature, i.e. a novel feature^34^ resulting from *de novo* or somatic gene mutations that are species specific leading to changes in protein function and/or its expression. An alternative idea could be that it is a stochastic phenomenon resulting from an accumulation of *de novo* mutations that are lost without evolutionary selection. This would explain why regenerative capacity is not widespread even among the same species. For a review discussing the molecular mechanisms underlying different regenerative mechanisms, please see Slack et al.^34^

## Of mammalian brain and regeneration

Mammals are known to have an impaired capacity to regenerate despite ongoing homeostasis in certain tissues (e.g. the skin, intestine, and liver) with exception of the spiny mouse (genus: *Acomys*) discussed earlier. Intriguingly, studies in rodents show evidence for a latent regenerative potential in the central nervous system,^32–35^ which is however limited. An increasing number of studies in rodents suggest that quiescent NSCs can activate and proliferate following injury^32^^,^^36–40^ (Table 1) and that the regenerative capacity of the adult brain can be stimulated with growth factors and small molecules.^41^^,^^42^ Besides NSCs’ permissiveness to activation and proliferation, they need to be able to differentiate into the appropriate cell types and integrate into the local circuitry. Although some results in rodents show that NSCs (either endogenous or following transplantation) have the potential to develop into the correct neuronal subtypes that form the appropriate connections,^43–45^ other data show inefficient differentiation and long-term survival of newborn neurons.^10^^,^^46^

**Table 1.** Overview of neurogenic and regenerative capacity over different species.

Species	Adult neurogenesis	Sites of adult neurogenesis	Cell types implicated in neurogenesis	Brain regeneration in adults?	Gliosis/scarring after brain injury?	Role of inflammation	Citations
**Zebrafish**	Yes	Widespread (pallial and subpallial ventricular zones, ventricular niches)	Radial glial cells, Neuroblasts	Yes	Minimal scarring; glia support regeneration	Transient inflammatory responses promote regeneration	PMID: 21595047; PMID: 22028327; PMID: 16682018; PMID: 20155821; PMID: 11978945; PMID: 22007133
**Newts/salamanders**	Yes	Widespread (telencephalon, ventricular/subventricular zones, and other brain regions)	Ependymoglia (radial glial cells)	Yes	Minimal scarring; glia support regeneration	Transient inflammatory responses promote regeneration	PMID: 36048956; PMID: 27156560; PMID: 24749074; PMID: 21068061; PMID: 36048929; PMID: 17611231
**Rodents**	Yes	Subgranular zone of the dentate gyrus of the hippocampus and the subventricular zone aligning the lateral ventricles	Neural stem cells, transit amplifying progenitors, neuroblasts	Some functional recovery driven by plasticity; no tissue regeneration	Reactive gliosis and glial scarring	Acute inflammation can transiently enhance neurogenesis, but chronic inflammation impairs progenitors and promotes scarring	PMID: 12466205; PMID: 27647730; PMID: 24811379; PMID: 24090877; PMID: 19332781; PMID: 12031271; PMID: 11784788; PMID: 10500226; PMID: 29439238; PMID: 39762661
**Spiny mouse**	Yes	Subgranular zone of the dentate gyrus of the hippocampus and the subventricular zone aligning the lateral ventricles; increased neurogenesis vs rodents	Neural stem cells, transit amplifying progenitors, neuroblasts	Functional recovery after stroke driven by neural plasticity; no tissue regeneration	Reactive gliosis and glial scarring	N/A	PMID: 33277722 PMID: 39706830
**Primates**	Yes, reduced vs rodents	Subgranular zone of the dentate gyrus of the hippocampus and the subventricular zone aligning the lateral ventricles; lower than in mice; more pronounced in younger animals	Neural stem cells, transit amplifying progenitors, neuroblasts	Limited (no true structural regeneration; functional restoration through neuroplasticity)	Reactive gliosis and glial scarring	Similar to humans	PMID: 10220454; PMID: 10521353; PMID: 34798047
**Humans**	Very limited to absent	Low, age sensitive in subgranular zone of the dentate gyrus of the hippocampus and subventricular zone aligning the lateral ventricles	Neural stem cells, transit amplifying progenitors, neuroblasts	Limited (no true structural regeneration; functional restoration through neuroplasticity)	Reactive gliosis and glial scarring	Inflammation associated with scarring and neurodegeneration	PMID: 40608919; PMID: 33762407; PMID: 29513649; PMID: 31130513; PMID: 30911133; PMID: 29625071; PMID: 41741649; PMID: 25621867; PMID: 23746839

N/A = not applicable.

The current dogma postulates that quiescent NSCs of the adult and aged human brain have an impaired capacity to activate and regenerate the brain following injury. Yet, a few studies in humans suggest that NSCs activate and proliferate in the SVZ after injury. Increased NSC proliferation has been reported in the SVZ following stroke^47^ and Huntington’s disease,^48^ and SOX2-positive NSCs have been shown to increase in the human SVZ after traumatic brain injury.^49^ In contrast, ^14^C birth-dating, a technique used to identify newborn neurons, showed no evidence for significant neuronal replacement in Huntington’s disease.^10^ These studies show that the role of adult neurogenesis in mediating brain repair in humans is impaired. However, it should also be emphasized that studying neurogenesis in the adult and aged human brain following injury is technically challenging. It is important to keep in mind that our current knowledge of the capacity of the human brain to replace lost neural cells is limited to the end stage of neurodegenerative diseases or injury and reflects the NSC activation state decades after initial neural loss. How NSCs respond to injury or neurodegenerative diseases at early stages remains unknown. Scarcity of high-quality post-mortem brain tissue, especially at younger ages and at early stages of neurodegenerative disease or injury, limits our understanding of the capacity of the human brain to repair. The fact that we find little evidence for NSC proliferation or differentiation does not necessarily mean that NSCs do not proliferate in the initial stages of neural cell loss.

## Keeping neural stem cells in check: an evolutionary brake

So why is the capacity of the human brain to regenerate inefficient, despite the presence of a potential stem cell source to regenerate the brain? According to the theory of adaptation to the environment discussed earlier,^31^ for tissues and organs that undergo recurrent injury such as the skin, liver, and the intestinal epithelium, it would have been evolutionary beneficial for species’ survival to be able to regenerate those tissues. However, humans are, compared to most species, relatively long lived, and therefore, tissue homeostasis may present disadvantages as uncontrolled differentiation under homeostatic conditions would be more deleterious and significantly increase the chance for tumor formation. Indeed, organs and tissues such as skin epidermis, liver, and intestine epithelium are prone to tumor formation. Therefore, shutting off the production of new cells may be beneficial for long-lived species such as humans, as a brake to prevent tumor formation.

The quiescent NSC state may not only be a mechanism to prevent NSC depletion in the mammalian brain but also to limit NSCs becoming malignant in long-lived species. Although it is still unclear whether NSCs from the SVZ could be a source of tumor cells,^50^ several gene mutations are known to promote oncogenic changes in NSCs resulting in increased proliferation. Indeed, combination of mutations in *p53*, *Pten*,^51^ and *Nf1*^52^ in NSCs was shown to result in the generation of glioma and astrocytoma cells in the mouse. These tumor suppressor genes are also often mutated in primary glioblastoma in humans,^51^ resulting in decreased expression of these tumor suppressor genes. Downregulation of *p53*, *Nf1*, and *Pten* in NSCs induced proliferation, increased self-renewal, and decreased astrocytic differentiation potential in mice.^52^^,^^53^ Cell cycle genes *Cdk4* and *Cdkn2a* were shown to be upregulated following inactivation of *Pten* and *p53* in mice.^54^ Mutations in *p53* and *Pten* stimulate an undifferentiated state with high renewal potential.^51^ A previous study showed that downregulation of *PTEN* expression in human NSCs result in changes in gene expression and metabolic profiles reflecting neoplastic cells.^55^ These changes are mediated by *PAX7*, which was shown to be upregulated in human glioblastoma cells lacking *PTEN* expression and promoted oncogenic changes in human embryonic stem cell–derived NSCs.^55^ In high-grade glioma patients, *TRIM11* was shown to be overexpressed in glioma stem-like cells and to promote proliferation mediated through the epidermal growth factor receptor (EGFR).^56^

The expression of tumor suppressor genes and oncogenes in brain tumors was shown to also be regulated by miRNAs.^57^ For example, in a human glioblastoma cell line, overexpression of *REST* promoted stemness,^58^ through the miR-21 gene, which is increased in human glioblastoma cells^59^ and is a repressor of *NANOG*, *SOX2*, and *OCT4*.^60^ MiR-34a expression is downregulated in human glioma cells and in *P53* mutant glioblastoma cells.^61^ Enhanced expression of miR-34a was shown to induce apoptosis, G2 cell cycle arrest, and senescence in human medulloblastoma cell lines.^61^ Increasing miR-34a expression downregulated Notch1/2 signaling, thereby inhibiting stemness in glioblastoma cells.^62^ MiR-34a also reduced the expression of the Notch ligand Delta-like 1 resulting in decreased stem cell proliferation, increased apoptosis, and neuronal differentiation in mouse tumor cells.^63^ Interestingly, miRNA expression profile was shown to differ between quiescent and activated NSCs in the mouse SVZ and to regulate NSC proliferation and lineage commitment.^64^ In contrast, species like the axolotl are not only highly regenerative but also seem to be resistant to cancer. Newts and salamanders, including axolotls, are regenerative but seem to have a low incidence of tumors, although more thorough and systematic studies are needed to convincingly determine this. The red spotted newt, which has extensive regenerative capacity, was shown to re-activate quiescent EGCs and replace lost dopaminergic neurons in a Parkinson’s disease model.^65^ The authors showed that quiescent NSCs activated in response to decreased dopamine levels and that administration of L-dopa inhibited this neurogenic response. This has been proposed as a negative feedback mechanism to prevent tumor formation. In the axolotl, quiescent ependymoglia were shown to proliferate and replace lost neurons by activating a regeneration specific transcriptional state and initiating a regenerative neurogenesis program, which is very similar to homeostatic neurogenesis.^28^ One study also suggested that ependymoglia cells repair injury to the telencephalon through the expression of an olfactory cue, which remains to be identified.^66^ Future studies in axolotls and newts should address whether there is reduced tumor formation in their brain and the safeguards against oncogenic transformation.^26^^,^^67^ Altogether this highlights that besides the capacity to initiate a regenerative program, the brain needs a brake mechanism to shut it down to prevent NSC depletion and tumor formation.

How can we reconcile these apparent contradicting views with on the one hand that increased NSC quiescence may act as a brake to prevent NSCs from going rogue and on the other hand that highly regenerative species do not necessarily have a higher tumor incidence? Perhaps this is due to stochasticity and genetic drifting rather than adaptive mechanisms. One could speculate that some species got lucky in the genetic lottery and express a combination of gene regulatory networks that support regenerative processes and prevent the formation of tumors. More loss- and gain-of-function studies in *in vivo* models are needed to determine whether a mechanistic link between regeneration and tumor formation exists.

## A question of time and place

Another alternative theory is that regional and temporal processes regulate regenerative potential. For example, the idea that cancer is a wound that never heals. This idea was first suggested by Virchow more than 150 years ago and later articulated by Dvorak.^68^^,^^69^ It is rooted in observations, many now experimentally confirmed that the tumor and wound microenvironment activate many of the same processes including inflammation, proliferation, migration, and angiogenesis. Interestingly, these processes do not resolve or are exacerbated in tumors.^68^^,^^69^ It also aligns well with an emerging body of literature that suggests that traumatic head injuries may predispose to develop brain cancer.^70^

Yet the picture is more nuanced. If we look at neurodegeneration as a chronic non-healing injury, it does not fit the framework that head injury could predispose to tumor formation. In fact, it has been observed that patients with neurodegenerative diseases like Alzheimer’s have a reduced risk of developing cancers, including glioblastoma.^71^^,^^72^ It has also been proposed that this anticorrelation may be due to distinct etiologies, for example, Alzheimer’s disease is associated with increased expression of the tumor suppressor gene *P53* which may exert a protective effect against cancer.^71^ A better understanding of the molecular similarities and differences between neurodegeneration and traumatic brain injury, which may be characterized as chronic or acute injuries, and their respective links to cancer risk and early oncogenic processes would help to delineate the relationship and important targets for intervention in these processes. It also underlines that both the brain environment and intrinsic cellular characteristics likely regulate the balance between disease development (i.e. neurodegenerative disease and tumor formation) and regenerative capacity.

But what regulates the capacity of NSCs to regenerate? Besides intrinsic molecular factors, such as specific signaling pathways, extrinsic factors in the environment are also known to inhibit NSC activation and regenerative processes. In mammalian species including humans, following severe injury, a so-called glial scar is formed. This glial scar is composed of reactive astrocytes, microglia, and several extracellular matrix proteins that form a border along the lesion site.^73^ Glial scar formation is an important mechanism to constrain the lesion site, yet it does not resolve. Several studies have shown that the expression of extracellular matrix proteins in the glial scar prevents axonal growth, cell migration, and remyelination.^74^ However, work by the Sofroniew lab demonstrated that preventing glial scar formation increases the lesion size and does not promote regenerative processes.^75^ Importantly, composition of the injury site, scar, and extracellular matrix varies between brain regions and lesion types, which determine the severity of the glial scar formation (Table 1).^76–78^ Interestingly, as we have seen, glial scar formation in the spiny mouse brain is attenuated, and both amphibians and teleost fish lack gliosis^67^.

This raises many questions concerning the intrinsic and extrinsic molecular mechanisms that trigger and regulate regenerative processes and underlines an important role of the injury environment in mediating successful regeneration. In this regard, the human brain has to deal not only with potential traumatic injuries but also with other forms of brain damage, namely neurodegeneration. This type of damage is progressive, diffuse, and results from pathological changes in the brain. It remains unclear if NSCs are affected by the disease pathology as well and how this would affect their regenerative response. The pathological environment likely hinders repair as one pathological hallmark of many neurodegenerative diseases is increased inflammation, which was shown in rodents to maintain NSCs in a quiescent state.^16^^,^^79^ Yet, inflammation induced by a stab wound injury but not by lipopolysaccharide (LPS) treatment (mimics a viral-like infection) was shown to be essential for newborn neurons to properly integrate into the local circuitry in a mouse model. Stab wound injury was shown to activate the complement pathway, synapse pruning, and increase the expression of Gfap and Vimentin. Hence, understanding how changes in the environment, that is extrinsic factors, can either be permissive or inhibitory to regenerative processes and affect NSCs’ regenerative potential could reveal targets that can be used to promote a more permissive environment to boost the regenerative potential of NSCs for successful brain regeneration.

Besides the role of environmental signaling in regenerative processes, other temporal and regional aspects may also influence the regenerative potential. For example, *Xenopus laevis,* or clawed frogs, are able to regenerate the brain in larval stages but not as adults. Studies suggested that although NSCs are still present in the brain of adult clawed frogs, they have an inefficient capacity to migrate and repair injury,^80^ suggesting that some species may have temporal specificity for their regenerative capacity. This could correlate to stem cell intrinsic mechanisms and/or inhibitory environmental cues. In the teleost fish, different stem cell sources have been described in different brain regions, which also differ in their regenerative capacity suggesting regional specificity for regenerative potential.^81^ While inflammation and gliosis were shown to inhibit brain regeneration in several species, including mammals and amphibians, it was shown to promote proliferation of stem cells and trigger neurogenesis in zebrafish.^82^^,^^83^ For a review comparing molecular mechanisms underlying regenerative processes across vertebrates, please see Alunni et al.^84^.

Studies in different species suggest that differences in the environment and in the interaction of NSCs with its niche significantly affect their capacity to regenerate the brain. By understanding the interplay of intrinsic and extrinsic mechanisms that regulate neurogenesis and regeneration in different species, one could identify targets to boost regeneration in the human brain to fully recover brain function.

## Future outlook

With increased aging of society, the disease burden caused by neurodegenerative diseases is expected to grow. Brain injuries caused by stroke or trauma also have a high incidence. The impact of cognitive and motor impairments caused by brain injuries and/or damage is extensive with yet no prospect of a cure. Although the human brain has an impaired capacity to repair, the fact that a NSC pool remains in the aged and diseased human brain offers a potential avenue for repair. As we have seen, it is still unclear whether regeneration in the human brain is an evolutionary relic or whether a latent potential exists that could be triggered if properly stimulated. Understanding how regenerators are fundamentally different from humans and what specifically allows species like the axolotl to promote brain regeneration could provide molecular targets to boost the capacity of the human brain to repair. Advances in (single-cell) omics techniques will offer novel insights into molecular mechanisms that could regulate the regenerative capacity of NSCs and increase the molecular resolution into interspecies comparisons.^85^^,^^86^ Current studies are looking into the potential of small molecules or miRNAs to boost the regenerative capacity of NSCs, while others are developing reprogramming approaches to stimulate regeneration. The rise of new techniques such as brain organoid models, fate mapping, clonal analysis, and reprogramming tools (e.g. Crispr-Cas9) will allow us to revisit long-standing knowledge gaps and old dogmas, such as the neurogenic potential of NSCs in diseased brains. Moreover, reprogramming tools can help dissect the molecular switches needed to successfully induce regeneration. Combination of single-cell RNA sequencing cross species comparison and genetic manipulation will enable us to understand why some species have more efficient regenerative capacity than others.

This perspective highlighted several knowledge gaps and underscores the importance of interdisciplinary studies and cross species comparisons. We discussed studies in several species suggesting that differences in the environment and in the interaction of NSCs with their niche significantly affect their capacity to regenerate the brain. By understanding the interplay of cell intrinsic and extrinsic mechanisms that regulate neurogenesis and regeneration in different species, one could identify molecular targets to boost regeneration in the human brain to fully recover brain function. Studying the capacity of NSCs to regenerate the human brain is paramount to determine if regeneration in the aged and diseased human brain could be unlocked or whether it is an evolutionary relic.
